# “After torture, everything changed”: the unpacking of trauma from torture with interpretative phenomenological analysis and Merleau-Ponty’s theory of the lived body

**DOI:** 10.1186/s40359-025-02507-4

**Published:** 2025-03-09

**Authors:** Ana Carla S. P. Schippert, Ellen Karine Grov, Ann Kristin Bjørnnes

**Affiliations:** Institute of Nursing and Health Promotion, Oslo Metropolitan University, Oslo, Norway

**Keywords:** Torture, Interpretative phenomenological analysis, Merleau-Ponty, Retraumatization

## Abstract

**Background:**

Trauma from torture is expressed primarily through bodily sensations and emotions, reflecting its deep imprint on the body’s memory. Merleau-Ponty’s existential phenomenology, which emphasizes the lived body, provides a valuable framework for discussing the intricate interplay between physical and psychological experiences. Through this approach, we gain a deeper understanding of the profound impact of torture on survivors, which in turn informs holistic recovery strategies.

**Objectives:**

The primary objective of this study was to explore the nuanced experiences of individuals who have endured torture, aiming to cultivate a profound comprehension of their journeys. Additionally, this study sought to explore the inherent risks of retraumatization within healthcare settings.

**Methods:**

This study, approved by the Norwegian Committee for Medical and Health Research Ethics, involved in-depth interviews with six torture survivors. The recorded interviews were transcribed and analyzed using interpretative phenomenological analysis (IPA). The article explores survivors’ experiences by means of Merleau-Ponty’s theory of the lived body.

**Results:**

The study identified four main themes: resisting torture–ignoring the body; fear and vulnerability–the unsafe body; broken trust–the broken body; and resilience–rebuilding the body. The participants reported various torture methods, including physical violence, asphyxiation, dragging, electric shocks, witnessing torture and murder, and sexual abuse, along with positional torture. They also noted instances of health-related torture, such as the deliberate withholding of medical care and the involvement of healthcare professionals.

**Conclusions:**

Survivors’ accounts of torture raise awareness about its widespread impact and deepen the understanding of its physical and psychological effects. Merleau-Ponty’s concept of the lived body enhances our grasp of the body’s connection to the world, informing better care and retraumatization prevention. This perspective can shape public opinion, policy, and global efforts to prevent torture, support survivors, and improve healthcare, while personal stories humanize the issue and challenge torturers’ claims.

**Supplementary Information:**

The online version contains supplementary material available at 10.1186/s40359-025-02507-4.

## Background

According to Amnesty International, war, violence, and terrorism are daily occurrences in various parts of the world [1], resulting in pervasive human rights violations and torture on a global scale. The Norwegian Refugee Council (2023) reported that more than 100 million individuals have been forcibly relocated and are currently seeking refuge on a global scale. Many refugees, including those in Norway, have suffered political persecution, severe abuse, or torture [2–4] and face serious psychological and physical consequences.

The United Nations Convention Against Torture (1984) defines torture as “the deliberate infliction of severe suffering for purposes like information extraction, punishment, or coercion” [5]. Trauma from torture results from the severe and often enduring physical, psychological, and emotional harm inflicted upon individuals [6] causing both physical and psychological trauma [7]. The trauma resulting from such experiences is multifaceted and profoundly impacts an individual’s overall well-being [6].

The impact of torture is encoded in the body and sensory systems, and the interplay between embodied cognition [8] and the recollection of traumatic experiences manifests in bodily sensations and emotional responses rather than verbal expression [9]. Torture resulting in severe physical and psychological harm renders the body a site of trauma [10].

The significant impact of torture and its lack of acknowledgment within society and clinical contexts increase a survivor’s vulnerability and the risk of retraumatization [11–14]. Retraumatization during healthcare has been described by survivors [11, 15] and recognized by healthcare professionals [16].

Healthcare professionals play a vital role in supporting the recovery of torture survivors, making it essential to understand the interplay between psychological distress and physical symptoms to minimize the risk of retraumatization in clinical settings [11, 17]. Retraumatization refers to the distress caused by re-experiencing traumatic stress triggered by situations, environments, or treatment interactions, which evokes past trauma, leading to significant emotional and physical responses such as flashbacks [18]. As the trauma of torture stems from the infliction of extreme physical pain, retraumatization can occur in healthcare settings when the body is manipulated, particularly during procedures that cause pain. This retraumatization may arise when medical examinations, treatments, or procedures inadvertently replicate sensations or experiences reminiscent of the original trauma [15].

For survivors of torture, even routine medical procedures can evoke intense psychological distress, flashbacks, or panic, potentially leading to retraumatization [11, 15]. To reduce the risk of retraumatization, it is imperative to recognize these potential triggers and ensure that patient care is conducted with sensitivity and an understanding of the survivor’s unique needs [16, 19]. Many healthcare providers, especially those in somatic departments, are often unaware of the risk of retraumatizing patients who have experienced torture [11, 20]. While the concept of retraumatization is mentioned in the literature, it is often accompanied by an insufficient explanation of the underlying mechanisms or processes involved [15]. Theories like Merleau-Ponty’s existential phenomenology provide valuable insights into the process of retraumatization after torture. By emphasizing the interconnectedness of the mind, body, and environment, Merleau-Ponty’s framework helps us understand how survivors relive trauma through embodied experiences, in which sensory triggers or environmental cues can reactivate the physical and emotional pain of past torture. This perspective highlights the importance of addressing both the psychological and embodied aspects of trauma to prevent retraumatization and support holistic recovery [21].

### Merleau-Ponty: perception through the body

Merleau-Ponty argues that individuals experience and interpret the world through their physical states, which are closely linked to mental processes [21]. Contrary to Cartesian dualism, his philosophy views the body as central to perception, cognition, and identity, making it crucial for engaging with our surroundings [22]. This philosophy has influenced many fields, including neurobiology and robotics. Researchers like Wehinger (2024) argue that Merleau-Ponty’s phenomenological concepts are key to understanding how humans adapt to their environments [23]. Analyzing the mechanisms of perception and action has significantly advanced artificial intelligence and robotics, enabling systems to learn and adapt through physical interaction rather than relying solely on abstract computational models. These technologies exemplify the embodied connection between the body and the environment, a concept articulated by Merleau-Ponty, highlighting how cognition emerges through dynamic engagement with the physical world [24, 25]. Contemporary psychology has been profoundly influenced by Merleau-Ponty’s concept of the body as an active subject, particularly in the development of “embodied cognition.” Researchers such asZahavi (2020) and Gallagher (2022) argue that mental processes, including thought and perception, are deeply intertwined with physical interactions with the environment, emphasizing that conscious experience is rooted in the body’s physical experiences rather than confined to the brain [26]. This perspective is particularly relevant in the context of torture, where inflicted trauma disrupts the fundamental integration of the mind and body. Survivors often experience retraumatization in everyday situations, such as hearing loud noises that resemble the sounds of their captivity, encountering certain scents associated with the location of their torture, or being somewhere that mimics the conditions of their detainment [27, 28]. These triggers can reawaken embodied memories, causing intense psychological and physical distress and highlighting the pervasive impact of trauma on both the mind and body. Torture can deeply disrupt a person’s sense of self and reality, affecting both their physical health and overall experience of life [29].

To explore the experience of life, researchers often employ interpretative phenomenological analysis (IPA) [30, 31]. IPA is particularly effective for studying complex, deeply personal phenomena, such as trauma and retraumatization, because it focuses on the subjective meanings that individuals attach to their experiences.

### IPA and Merleau-Ponty

Incorporating IPA with Merleau-Ponty’s theory of the lived body offers a unique and relevant perspective for understanding torture trauma and retraumatization. IPA focuses on how individuals interpret their personal experiences, emphasizing their subjective realities [30], and Merleau-Ponty’s concept highlights the body’s central role in human experience and perception [32]. Together, they reveal that trauma is both a psychological and deeply embodied phenomenon. This approach shows how survivors experience torture and retraumatization through their bodies and how this affects their interactions, especially in healthcare settings. By integrating these frameworks, we gain new insights into the relationship between physical and psychological trauma, enhancing our understanding of how survivors navigate and cope with post-trauma realities. This perspective expands trauma research by shedding light on the embodied dimensions of suffering and healing. By integrating Merleau-Ponty’s phenomenology, psychologists and other healthcare professionals can gain a deeper understanding of the lived experiences of torture survivors, enabling them to provide care that is more empathetic, holistic, and effective.

### Aim of the study

The objective of the current study was to gain insights into the experiences of torture survivors. This study represents the third stage of a project [33] aiming to develop and evaluate guidelines for preventing the retraumatization of torture survivors during surgical care. This article explores survivors’ experiences of torture, interpreted and discussed through the lens of Merleau-Ponty’s theory of the lived body.

Merleau-Ponty’s theory provides a foundational framework for understanding embodied knowledge. In this study, the patients’ sense of embodiment (corporeality) is profoundly disrupted by their experiences of torture, which significantly impact and shape their lifeworld, including their lived relations, lived space, and lived time [34, 35].The study examines how survivors perceive and relate to their own bodies after torture, shedding light on the profound interplay between physical and psychological trauma.

IPA emphasizes a deep understanding of each participant’s lived experience. By focusing on a smaller number of participants, researchers can conduct thorough, detailed analyses that capture the complexity and nuance of individual experiences. This study’s idiographic approach prioritizes examining individual cases in detail before drawing broader conclusions [30, 36].

## Methods

### Recruitment

The study design and recruitment strategies have been published previously [11, 33]. This article is based on in-depth interviews with six torture survivors. The sample size aligns with the recommendations of IPA [36, 37], which suggest including six to eight participants. The final sample size was determined by the richness and depth of the data provided by each participant [30, 38].

**Fig. 1:**
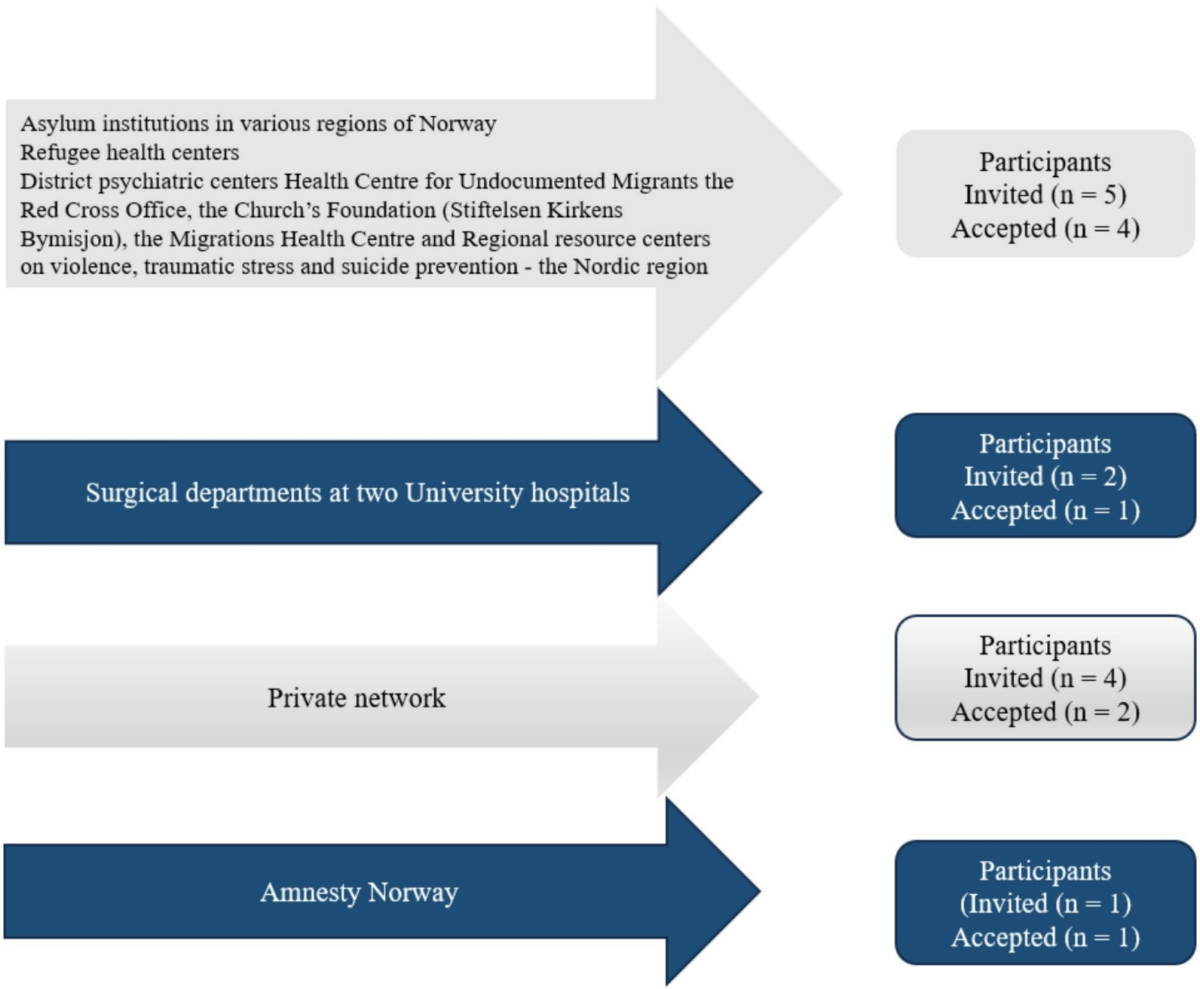
Recruitment strategy

A broad recruitment campaign was carried out across healthcare institutions and organizations working for refugees. Collaboration with various organizations enabled the dissemination of research information, with professionals from these institutions identifying potential participants who agreed to share their experiences of torture. Participants were recruited based on their comprehension of the study and their ability to consent [11]. The study followed ethical guidelines [39] and was approved by relevant authorities (i.e., Regional Committee for Medical and Health Research Ethics (#227624) (Supplemental file 1), the data protection official for research, and the data protection officer of Akershus University Hospital). Confidentiality was ensured, and participants signed consent forms (Supplemental file 2). Figure 1 shows the recruitment strategy.

### Interviews

Semi-structured interviews began with demographic questions and transitioned to discussions about participants’ torture experiences. The format was flexible, allowing for real-time adjustments and follow-up questions based on responses [40]. Participants were informed that the purpose was to explore their experiences of torture and the impact these experiences had on their lives. The interviews could be terminated at any time by the participants.

The interview guide, shown in Supplemental file 3, was pretested with colleagues to reduce the risk of bias, assumptions, confusing language, and specialized terms. Interviews were conducted by the first author between May 2021 and January 2023 in Norway in settings chosen by the participants. Interpreters were made available to all participants; however, only one participant chose to use one. The remaining interviews were conducted in Norwegian. Additionally, one participant requested the presence of a familiar person for support.

The interviews, lasting 1–2.5 h each, were recorded and stored securely in accordance with the guidelines of the Norwegian Centre for Research Data and Oslo Metropolitan University. Six of the eight originally recruited participants discussed their torture experiences.

The research team implemented a comprehensive protocol to support participants in managing intense reactions during or after the interviews. Specialists in bereavement, trauma, and stress were available for follow-up care as needed. To ensure participants’ well-being, the interviewer conducted follow-up checks one day, one week, and one month after the interview. Participants were also encouraged to reach out to the research team if they required additional support. While one participant contacted the interviewer twice, it was solely to provide additional information. Emotional support was readily accessible, and follow-up conversations or guidance were provided if significant reactions arose after the interview. To protect the participants’ identities, pseudonyms were used throughout the dissemination of the results.

Transcriptions from the interviews were analyzed using IPA [36–38]. The analysis aligns with the phenomenological perspective outlined by Merleau-Ponty, which serves as a framework for discussing the findings.

### Assurance of quality measures

In this study, phenomenology was used to focus on the participants’ experiences. Using IPA’s double hermeneutics, the researchers interpreted narratives and emotions arising from experiences of torture [30, 37]. The IPA methodology encourages a reflexive approach, acknowledging the subjective realities of researchers [41]. Ongoing reflection helps ensure that researchers’ biases do not overshadow participants’ perspectives.

### Participants

The study involved torture survivors from diverse countries in South America and the Middle East. These individuals had been living in Norway for periods ranging from 7 to 40 years and had received treatment in various somatic departments across the country, including surgical units. A description of the participants is presented in Table 1.

**Table 1:** Description of the participants who disclosed their torture experiences

Participant 1 (P1)	Participant 2 (P2)	Participant 3 (P3)	Participant 4 (P4)	Participant 5 (P5)	Participant 6 (P6)
***“They came at night. Every night!”***	***“Then they hung me by my legs and put me in water.”***	***“There was hunger***,*** there was fear***,*** and there was anxiety. All the time!”***	***“I ran and I ran***,*** and I hoped that I would be shot from behind while I was running. But they caught me again.”***	***“Torture***,*** it is just suffering! When you start bleeding under torture…that’s just the beginning!”***	***“The most agonizing part was hearing the screams of women being tortured while their children were present. Today***,*** I can’t stand it when someone screams.”***
P1 is approximately 60 years of age and has resided in Norway for more than 30 years after being granted asylum in the 1990s. He was imprisoned in his home country when he was just 17 years old for his political activity. During his two years of imprisonment, he was subjected to various methods of torture in a systematic way. In Norway, he attended education and became a professional in the health sector, where he worked for many years. Throughout his life in Norway, he became increasingly involved in humanitarian work, helping countries with scarce resources. He was married several times and had several children. His passion for cooking created opportunities to gather good friends and family around him.	P2 is more than 60 years old and has been living in Norway for over 30 years with his wife and children. He was politically active in his home country and became imprisoned for it when he was about 20, when he was already married and had a child. He was granted asylum in Norway in the 1990s. During 2.5 years of imprisonment, he was subjected to systematic torture. When he arrived in Norway, he was offered therapy with a psychologist, which he accepted. Despite encountering many challenges later in life, this remained the only contact he had with a psychologist. He devoted his life to caring for and supporting his children.	P3 is over 65 years old and was granted asylum in Norway in the 2000s. He is originally from the Middle East. He was exposed to violence and ill treatment by members of the military forces during war conflicts in his home country. He is a father of five and lives in Norway with his wife, daughter, and son. His health became very poor due to violence and ill treatment during the war. He appreciates the freedom in Norway and loves to go for walks despite the pain in his body.	P4 is originally from the Middle East. He was granted asylum in Norway in the 1900s after a two-year stay in prison in his home country, where he was systematically tortured by members of the military forces. He has never married and lives alone. He has been in contact with the healthcare system many times due to health problems related to torture. He likes to swim to forget his painful experiences.	P5 was born in the Middle East and became politically active when he was around 20 years old. At that time, he was tortured by members of the military forces, and in two years in prison, he was moved several times. He was subjected to torture in several of the prisons he stayed in. After two years, he fled to a neighboring country, where he was also tortured. In the 1900s, he was granted asylum in Norway. He never married and has no children. In Norway, he pursued higher education and has been working in the healthcare system. At one point, he was offered therapy by a psychologist, but after two sessions, he gave up the treatment. He loves dancing to forget his pain.	P6 was born in the Middle East and became politically active at age 35. During imprisonment, he experienced torture at the hands of military personnel. Over five years spent in incarceration, he was subjected to multiple transfers between correctional facilities and tortured during his confinement in different prisons. After five years in prison, he was granted asylum in Norway. He has offspring, has pursued educational endeavors, and devotes his leisure time to assisting other individuals who have been affected by political conflicts and human rights violations.

## Results

All individuals involved in the study reported having been subjected to various methods of physical and psychological torture. Five of the participants reported being subjected to repeated acts of torture during their detention, while one individual had experienced torture during his time in a refugee camp and had also been exposed to war-related circumstances in the past. The physical torture in detention facilities involved various methods of inflicting pain and suffering. Extended and random beatings were described, including the falanga method, which involves striking the planta pedis and results in severe swelling and tissue damage. Additionally, the participants described being subjected to submersion in water or detritus, as well as different forms of electricity, suspension, and blows to the head and body. They recounted starvation, poor prison conditions, extreme temperatures, and denial of medical treatment. Psychological torture commonly involved techniques aimed at inducing shame, guilt, and fear in the victims, including threats of violence against the victims or their families, simulated executions, sexual torture, and the deliberate deprivation of sensory stimuli. Four participants reported the involvement of healthcare professionals in their torture.

**Fig. 2:**
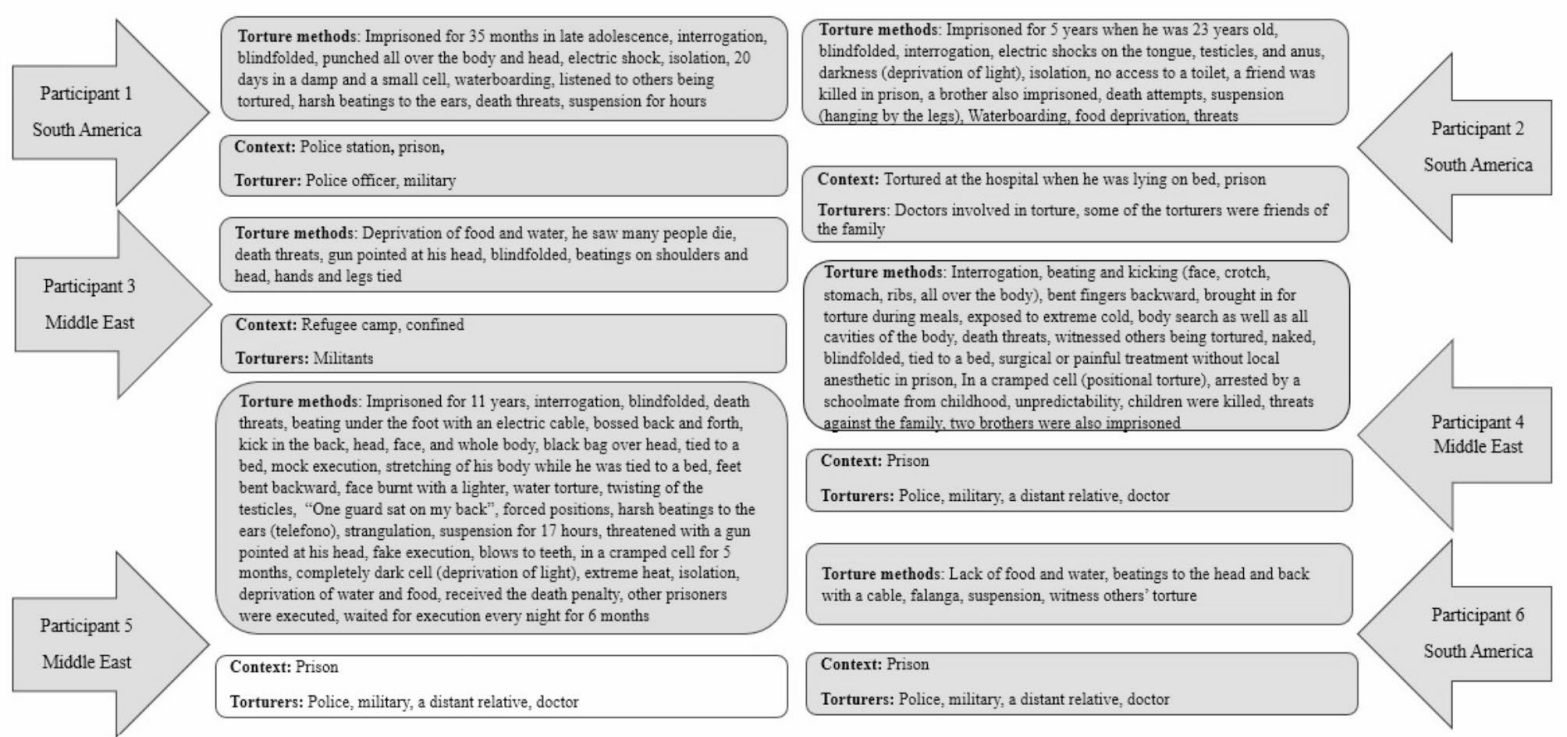
Presentation of torture methods

Figure 2 provides a comprehensive list of the torture methods the participants were subjected to and the contextual background.

The data analysis was iterative and manual. The primary author initially coded each transcript, refining the themes with subsequent transcripts to capture the underlying meanings. After coding, the primary author used a reflexive approach before collaborating with coauthors to verify themes and reach consensus. To strengthen inter-rater reliability, one author (AKB) read the quotes and checked the generation of themes throughout the entire analysis process. The independent evaluations by different researchers consistently identified the same constituent and superordinate themes, demonstrating inter-rater agreement and underscoring the rigor of the analysis [41, 42].

We aimed to understand the participants’ torture experiences by identifying common themes and condensing texts to capture the essence of their lived experiences. Three analysis cycles refined the data into central meanings. In the final cycle, the participants’ responses were distilled to 1–3 words to encapsulate core essences [43]. Interpretative statements and summative descriptions were annotated in the margins. Themes were generated by grouping interpretative statements and identifying connections. Diagrams of themes were created, and themes were merged as the analysis progressed. After individual transcript analysis, a cross-case analysis identified common themes, with corresponding sections categorized accordingly. The analysis of patterns and relationships led to new overarching themes. Circular interpretation refined final themes, categorizing them into major (superordinate) and minor (subordinate) components [37].

**Fig. 3:**
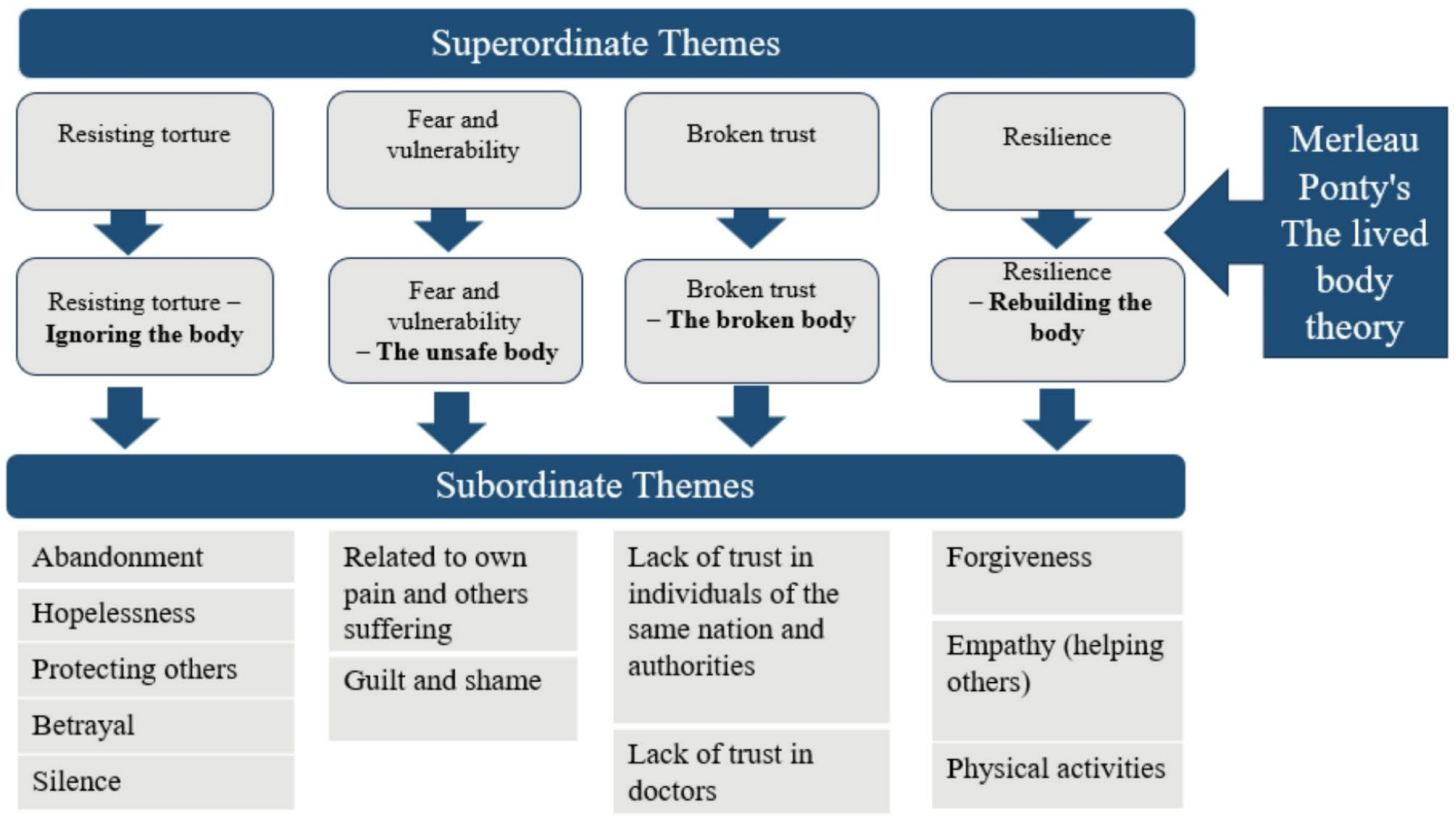
Summary of themes

The interview data analysis resulted in the identification of four superordinate themes and twelve subordinate themes. The four superordinate themes—resisting torture, fear and vulnerability, broken trust, and resilience—were reinterpreted through the lens of Merleau-Ponty’s theory of the lived body, leading to their renaming as: resisting torture–ignoring the body; fear and vulnerability–the unsafe body; broken trust–the broken body; and resilience–rebuilding the body. These reinterpretations align each theme with key aspects of the lived body as conceptualized by Merleau-Ponty. In the discussion section, the themes are further explored and analyzed in the context of Merleau-Ponty’s theoretical framework, providing a deeper understanding of the embodied nature of trauma and recovery. The themes are presented in Fig. 3.

### Resisting torture—ignoring the body

All the participants described diverse methods of torture employed to elicit information or confessions about opposition parties or terrorist groups. They reflected on how the pain inflicted on the body was the primary means by which the torturers attempted to achieve their objectives.

Participants described the necessity of consciously suppressing their bodily sensations to endure the pain and withhold information during torture. The following excerpt exemplifies how the overwhelming pain compelled them to adopt this strategy as a means of survival.

*I didn’t know where I was in pain. My back*,* my head*,* my arm…everywhere! So*,* I just…kind of…ignored the body.* P5.

Torture appears to disrupt the body’s natural prereflective harmony, forcing individuals into a heightened and agonizing awareness of their physical state. This shift in bodily awareness suggests that overwhelming pain does not merely cause suffering but also fundamentally alters the victim’s relationship with their body and self. The participants’ accounts indicate that their conscious attempts to suppress or detach from their bodily sensations can be interpreted as a strategy to reclaim control in an otherwise powerless situation. This effort to resist the pain and maintain autonomy over their awareness appears to reflect an act of defiance against the torturers’ intent to dominate and extract information.

*My primary purpose was to resist ceding and disclosing information to the torturers*,* which I successfully achieved by ignoring the pain.* P1.

This quote highlights the intense struggle survivors may face in coping with and resisting pain.

#### Abandonment: the insignificant body

The participants’ accounts suggest that torture not only physically isolated them from the outside world but also profoundly disrupted their relational sense of being with others, reducing them to a state of insignificance. The actions of the torturers appear to dehumanize them, stripping away their sense of identity and reducing them to mere objects of inflicted pain. One participant vividly expressed feelings of abandonment and insignificance, likening his experience to that of an animal:

*They treated me like an animal…even animals aren’t treated that way!* P4.

After enduring extremely painful torture methods, survivors described being left to endure their suffering in isolation. They shared how the torturers exhibited a profound lack of empathy, intensifying their feelings of abandonment and worthlessness. This absence of compassion seemed to leave participants with a deep sense of disconnection from the outside world.

*After the violence*,* I was left alone to die—without water*,* without food*,* and in pain.* P3.

Other participants described how the torturers showed complete disregard for the body’s natural reactions to pain and distress. It appeared that the pain, wounds, and blood were insignificant to them.

*The blood*,* the pain*,* they did not care! They just keep going!* P2.

Participants also recounted being subjected to isolation as a method of inflicting harm. This physical isolation seemed to evoke profound emotional and psychological feelings of loneliness and abandonment. One participant shared how, even years after his release, he continued to feel a deep sense of isolation and anxiety about being alone.

*Although they let me go*,* I refrained from going. I was at a loss for where to go and who to contact.* P5.

This quote illustrates how torture severs the victims’ connection to the outside world and dismantles their social relationships.

#### Hopelessness: longing for release from the confines of the flesh

The participants’ accounts revealed a pervasive sense of dread about their future during imprisonment, marked by a deep-seated belief that their end was near. Witnessing the executions of fellow captives reinforced this sense of inevitability, leaving them in a state of anxious resignation, merely awaiting their own fate. This context fostered a complex ambivalence toward life and death—on one hand, a yearning for release from relentless physical and emotional pain, and on the other, an instinctual fear of death.

Torture profoundly disrupted the survivors’ sense of agency, leaving them feeling powerless and confined within their own bodies. This entrapment within a physical form dominated by pain and trauma highlights the psychological toll of torture. For some, death emerged as a perceived escape—the only conceivable relief from their suffering. The longing for death was not merely an end to life but a desperate hope for liberation from the unrelenting torment imposed on them.

*While I was escaping and running away*,* I had the hope that they would shoot me from behind.* P4.

Participants explained that the initial release of blood during torture just marked the beginning of persistent suffering, leading to a profound sense of hopelessness and uncertainty about survival.

*Having begun to bleed was only the beginning. It was evil!* P3.

Torture can cause a breakdown of the self, as intense and uncontrolled pain fragments the victim’s identity, leading to feelings of disownership and existential disintegration.

#### Protecting others: offering their own body

The participants explained that their decision not to confess was primarily driven by allegiance to their political ideology and a desire to resist their torturers. However, they also appeared to recognize that the regime relied heavily on confessions to secure criminal convictions and understood that withholding confessions could increase their chances of eventual release. Additionally, confessing would likely exacerbate the torture, not only for themselves but also for others.

*It would have been even more torture if I had confessed.* P1.

Despite their immense suffering, all participants recounted situations in which they demonstrated profound empathy and compassion for others who were vulnerable to torture. These acts reflected remarkable bravery and altruism as they willingly exposed themselves to greater risk to protect others. Five participants described their refusal to confess or divulge information, motivated by loyalty to their comrades.

*I maintained my resolve and refrained from divulging any information regarding the individuals involved in our movement. I excelled in that particular task!* P1.

Two participants shared harrowing accounts of their siblings being tortured, some even to death, and expressed their own willingness to endure further torture, pain, or even death to protect their families. Their narratives suggest that a deep, unwavering desire to safeguard their loved ones served as a powerful motivator, strengthening their resolve to resist.

*They took my brother! He had done nothing! He wasn’t even 18 at the time. He was only a child!* P6.

One participant wept as he recounted the profound impact his imprisonment and torture had on his mother, who became gravely ill as a result. This highlights the immense suffering endured not only by the victims but also by their families. The participant revealed that his mother ultimately passed away from heart-related complications, which he believed were caused by her overwhelming sorrow. His narrative suggests a deep sense of grief and guilt over his inability to protect her from the consequences of his ordeal.

*The torturers*,* in a way…they also killed my mother*,* and I couldn’t help.* P4.

Another participant recounted his wife weeping as a result of the injuries he sustained during the torment, highlighting the immense agony survivors experience over the suffering endured by their families.

*Witnessing my wife cry was even more agonizing than my own pain; it shattered my heart!* P2.

All the participants expressed a strong desire to protect others, which appeared to surpass any inclination toward revenge. They recounted the profound impact of betrayal by once-trusted individuals, which seemed to deeply affect their sense of self and their relationships. Despite this, they remained steadfast in prioritizing the well-being of others. This unwavering commitment to safeguarding others, even at great personal cost, underscores their extraordinary compassion and resilience.

*One of the prisoners confessed and committed suicide. I was astonished! I had no idea he had thoughts like that. I did not know. I felt bad*,* even though I did my best to help him!* P1.

Even though the other prisoner had provided information that could harm others, the participant showed empathy and understanding.

#### Betrayal: the denied body

Complex emotional responses related to betrayal recurred in the participants’ narratives.

*A friend from school gave me over to the military. Now*,* I still find it difficult to understand that.* P1.

The participant expressed significant difficulty in coming to terms with a friend’s behavior, even after a considerable amount of time had passed. It appears that betrayal leaves a deep and lasting kind of sorrow. Another participant also described the impact of betrayal.

*I was betrayed by someone I trusted…but I would never do it myself…against others…no!* P2.

Despite recalling instances of betrayal, the participant consistently expressed a strong sense of duty to safeguard those around him, keeping his integrity and loyalty.

#### Silence: the invisible body

Five participants recounted struggling to maintain silence and withhold the information demanded by their torturers. They simultaneously tried to break the silence among fellow inmates, inquiring about information that could offer them protection.

*In prison*,* we had to get information from others. It was critical… the only way to keep us safe from the torturers. But we couldn’t say too much about ourselves.* P1.

Additionally, the participants commented on the silenced society that perpetuated secrecy surrounding torture.

*We were shuffled from prison to prison*,* ensuring that no one knew where we were. No one cared*,* and in the end*,* we were forgotten.* P5.

This quote illustrates the profound sense of abandonment, invisibility, and silence experienced by the participant. Being repeatedly moved from prison to prison not only physically isolated them but also deepened their perception that their suffering was unnoticed and that they had been forgotten by the outside world. This displacement was likely a deliberate tactic by their torturers to render them invisible, sever them from their communities, and erase their voices and stories. This appeared to reinforce the dehumanizing impact of being treated as if their lives held no value.

The participants also described how the silence surrounding their torture experiences continued even after their release. This enduring secrecy seemed to shroud their stories, making it uncommon for them to share their experiences openly. One participant had the courage to disclose his story but felt ignored and unheard.

*The psychologist*,* even though I talked about my story and my problems*,* ignored them and refused to help when I needed it!* P4.

The lack of societal support and discourse around torture may give survivors limited opportunities to share their experiences, even in secure environments like therapy sessions. The participants’ accounts suggest that their experiences of torture were met with silence and disbelief by society, which seemed to deepen their feelings of isolation and marginalization. This societal response diminished the gravity of their suffering and invalidated their lived experiences, potentially pushing them toward an existential crisis. The lack of acknowledgment by the broader community appeared to compound their trauma, making them feel invisible and further estranged.

This response may reflect society’s inability or unwillingness to confront the implications of the survivors’ experiences. In this way, the survivors’ torture becomes emblematic of their broader marginalization. Societal indifference not only exacerbates the psychological and physical wounds of survivors but also perpetuates their sense of alienation and disconnection from the world around them.

The participants also described difficulties related to being isolated from their families and society during their incarceration. Their difficulties were exacerbated by living in exile and lacking family in proximity. This in itself limited opportunities to discuss the past with family members, reinforcing the atmosphere of secrecy around their experiences of torture.

Two participants with partners and children described their decision to withhold their experiences of torture from their children. This choice appeared to stem from a desire to protect their families from the burden of such painful knowledge and their concern about how their children might react and the potential impact on their relationships. This dynamic reflects a complex interplay of protection, fear, and the deep emotional scars left by their experiences.

The act of withholding their stories can be interpreted as a coping mechanism to preserve family harmony and shield loved ones from the trauma they endured. One participant, overcome with emotion, explained this struggle while crying, illustrating the profound inner conflict and sacrifice involved in prioritizing his family’s well-being over his own need for expression and acknowledgment.

*Not even my adult children are aware of my past*. P1.

This quote demonstrates the enduring secrecy and silence survivors maintain about their traumatic experiences, even with those closest to them. It reflects the depth of their pain and the protective instinct to shield their loved ones from the emotional burden of their past. This silence also underscores the ongoing struggle survivors face in reconciling their personal histories with their current relationships as well as the isolation and unresolved trauma that can persist for years.

### Fear and vulnerability—the unsafe body

This theme encompasses the participants’ psychological responses to the acts of torture and other stimuli.

#### Related to their own pain and others’ suffering

All participants described psychological torture as more challenging to endure than the physical torture, and its long-term effects as more burdensome. One participant described his fear when the torturers approached.

*When they arrived with their heads covered and slowly walked around until they picked one of us*,* it was terrible! The one who was chosen*,* never came back.* P1.

This quote illustrates the psychological torment caused by the unpredictability and inevitability of violence. The mere act of selection created an atmosphere of dread and helplessness as those left behind were forced to anticipate their own potential fate. This narrative highlights the dehumanizing strategies of the torturers, designed to both harm individuals physically and instill pervasive fear and despair among those who remained.

Five of the participants expressed persistent feelings of fear despite residing in Norway, a country known for its secure environment. They remained apprehensive about the possibility of unexpectedly encountering their torturer.

*During the first period*,* I was obsessed with him. He did not torture me himself*,* but he gave orders to others. It turned out that these people were distributed throughout the entire world. I received a list with the names of everyone who was dispatched*,* and he was dispatched to Scandinavia. I hired a lawyer and was waiting for him.* P1.

One participant revealed that for four decades, he engaged in mathematical calculations to determine the age at which his torturer would likely no longer be alive. He had only recently attained a state of tranquility, understanding that the perpetrator of his torture was likely at least 80 years old and thus probably deceased.

The participants’ accounts suggest that torture had left them with a profound sense of insecurity and a loss of autonomy, fundamentally shattering their sense of safety and control over their own lives. This appeared to create a state of constant vulnerability and fear in which they felt perpetually threatened and exposed. This lasting psychological wound persisted long after the physical torture had ended. It manifested in hypervigilance, nightmares, and intrusive flashbacks that disrupted their daily lives and their abilities to maintain healthy relationships. The pervasive and enduring impact of torture shaped survivors’ emotional and relational worlds in profound and ongoing ways.

All participants described how their torturers’ absolute authority rendered them powerless to resist or protect themselves. Escalating pain and trauma heightened their vulnerability, leading them to question their ability to endure further suffering. They recounted how torture eroded their dignity as their treatment reduced them to the status of inanimate objects or animals.

*They likened me to pigs and other animals. They call me pig… Not even animals endure the same level of mistreatment that we did.* P4.

This quote illustrates how the dehumanizing treatment resulting from torture inflicted profound emotional distress, leading participants to view themselves as weak and worthless.

*I believed that I was strong. However*,* after the torture*,* everything changed.* P2.

Three participants described contemplating or even attempting suicide as a means of escaping further suffering.

*I considered suicide and looked for an object…which I did find…but it wasn’t sharp enough.* P1.

The participants’ accounts suggest that the experience of torture induced a profound sense of vulnerability, triggering deep personal transformation and resulting in lasting physical and psychological fragility. They perceived torture as intensifying their awareness of their vulnerability, which they expressed through their behaviors and emotional responses.

Two participants mentioned siblings who were also incarcerated, explaining that the presence of endangered family members further exacerbated their vulnerability during imprisonment. Additionally, the participants expressed apprehension about the potential repercussions of their incarceration and torture on their immediate family members and close friends, acknowledging that this increased their vulnerability.

One participant described remembering his brother’s pain and suffering as even more difficult than recalling his own torture. Another recounted that hearing the screams of others being tortured, especially women, profoundly impacted his sense of self, rendering him vulnerable to the suffering and distress of others.

*I can’t stand hearing other people scream…when little children scream on the subway*,* I…no no! It is too much!* P6.

This quote suggests that memories of torture remain vivid and deeply ingrained, even after many years, indicating a high likelihood of emotional and psychological reactions being triggered. It reflects the enduring impact of trauma and its capacity to resurface unexpectedly, influencing survivors’ daily lives.

#### Guilt and shame

The participants’ narratives also illustrate a profound internal struggle with feelings of shame. This emotional response appears to stem from the dehumanizing nature of their experiences, reflecting the deep psychological scars left by the torture and its impact on their sense of self-worth. When discussing sexual torture, two of the participants avoided precise terminology, instead using euphemisms such as “*they came under the cover of night*”P5. Similarly, they attributed conditions like hemorrhoids to torture without explicitly mentioning sexual assault. This reluctance to explicitly name their sexual torture indicates a deep sense of shame and discomfort. The participants’ use of euphemisms can be interpreted as a reflection of the profound intersubjective disruption caused by torture, suggesting that their experiences not only inflicted physical harm but also fractured their confidence in how they were perceived by others. The selective nature of their disclosures appeared to function as a coping strategy, enabling them to navigate and contain the intense shame associated with their bodily violations while maintaining some control over how their experiences were shared and understood.

The participants’ accounts revealed strong feelings of guilt, particularly tied to instances in which others were harmed while attempting to protect them. This guilt appeared to stem from a sense of responsibility and moral distress over the suffering of others.

*They alternated between punishing me and my lawyer. He…he didn’t make it. He took his own life…I couldn’t have known.* P1.

This quote highlights the feelings of helplessness and guilt experienced by survivors. It reflects their loss of control as their autonomy was stripped away, leaving them at the mercy of their torturers.

The guilt experienced by survivors can be interpreted as ingrained in their lived experiences, extending beyond their cognitive awareness to affect them at a profound physical and emotional level. This guilt appeared to permeate their sense of being, affecting how they related to themselves and the world around them. When the survivors witnessed or learned about the suffering of others, such as their lawyers or comrades, they seemed to internalize these events, transforming them into a deeply felt sense of responsibility and guilt.

### Broken trust—the broken body

This theme encapsulates the impact of torture on both the physical and psychological aspects of survivors’ lives, highlighting how the betrayal of trust mirrored the physical harm inflicted on the body. The participants reflected on the deliberate and calculated nature of their injuries, which they found especially distressing. Unlike accidental harm, the suffering they endured was the result of conscious, malevolent actions, which intensified their emotional and psychological impact. These experiences appeared to have profoundly disrupted their core beliefs about themselves and others. The deliberate infliction of harm challenged their positive self-perceptions, undermining their sense of agency and resilience. Moreover, the intentional betrayal of trust inherent in such acts deeply eroded their capacity to trust others, shaking the foundation of their interpersonal relationships.

The participants’ accounts suggest that the most devastating impact of their imprisonment and torture was the erosion of their trust in others. This loss of trust was a key consequence of their experiences and the psychological betrayal they endured. The deliberate and personal nature of the harm inflicted appeared to have disrupted their ability to rely on others, altering their perceptions of relationships and the social world. One participant’s reflections highlighted how this breakdown of trust became a central and enduring aspect of their trauma.

*During that time*,* the worst was not being able to trust others.* P1.

This quote emphasizes how the loss of trust became one of the most agonizing aspects of their experience, perhaps even more so than the physical pain. The betrayal and dehumanization inherent in their ordeal caused deep psychological and relational scars.

#### Lack of trust in individuals of the same nation and authorities

The participants described harrowing experiences of torture inflicted by members of their own community, compounded by betrayals from friends. These acts of betrayal damaged their ability to trust close relationships and disrupted the sense of connection and belonging that typically binds individuals to their communities. Consequently, the survivors often struggled to trust others from their home country, feeling a deep sense of insecurity in their presence. Instead, they found it easier to trust individuals from Norway, who had no ties to their country of origin. However, this trust remained fragile and did not extend to authoritative entities, such as the military police, even within the Norwegian system. One participant recounted an episode in which he had to share his story with the authorities while applying for compensation.

*I was doing well*,* but when I told the authorities about my story*,* everything turned dark.* P6.

#### Lack of trust in doctors

Four participants reported that their torture experiences involved the assistance of healthcare professionals. They expressed hesitancy in trusting medical professionals, often refraining from disclosing information. One participant spoke of his hesitation in sharing the full account of his torture experiences with healthcare providers.

*Should the physicians inquire about my experiences with torture*,* I would provide them with a selective account of the facts. However*,* not everything.* P2.

This can be interpreted as stemming from a deep-seated uncertainty about how healthcare providers might handle disclosed information. Survivors may fear that sharing their experiences could lead to judgment, misunderstanding, or even misuse of their personal accounts. This apprehension likely reflects the broader erosion of trust caused by their traumatic experiences, when vulnerability had been met with harm or exploitation. Their reluctance to disclose could also signify a protective mechanism to maintain control over their narratives and safeguard against further emotional exposure.

One participant recounted his interaction with a doctor who worked closely with the authorities during an assessment process to determine his status as a torture survivor.

*I answered the questions in a manner that would prevent the doctor*,* organizations*,* or any other secret police from abusing the information. I responded precisely to the inquiries that I intended to address. Nothing additional.* P1.

The participants’ reluctance to fully disclose their torture experiences to healthcare providers can be interpreted as a defense mechanism rooted in their history of betrayal. Their selective sharing of information reflects a lingering fear that their vulnerabilities might be exploited, echoing the profound mistrust caused by their traumatic encounters with complicit medical professionals. This indicates the psychological impact of their experiences, in which past betrayals had disrupted their ability to form trusting relationships with those in positions of care, perpetuating their sense of vulnerability and alienation.

The involvement of physicians in their torture seemed to provoke an existential crisis for survivors, deeply challenging their fundamental beliefs about human dignity, safety, and care. This resulted in a significant loss of trust in medical institutions and professionals. The survivors’ existential disintegration was intensified when healthcare providers—those entrusted with healing—became agents of harm. This made it profoundly challenging to rebuild trust in any healthcare setting.

### Resilience—rebuilding the body

This theme highlights positive adaptations and the capacity to maintain or restore mental and physical well-being following the experience of torture. It explores how survivors demonstrate resilience as they navigate the complex process of recovery and healing after enduring severe trauma. For this study, we adopted Van Breda’s (2018) definition of resilience, which identifies three essential components: adversity, outcomes, and mediating factors [44]. Van Breda conceptualizes resilience as a dynamic process that produces specific outcomes, with the relationship between adversity and outcomes profoundly shaped by mediating processes.

All participants reported that it took considerable time for their lives to regain a sense of normalcy following their experiences of torture. They highlighted improvements in their relationships, including greater openness, reduced isolation, reestablished trust, and more positive interactions with others. Additionally, they reported an improved quality of life, attributed to living in a secure environment, achieving financial stability, and experiencing reduced stress. Despite these advancements, they emphasized that this was an ongoing process and that they continued to work toward further healing and stability.

#### Forgiveness

All the participants recounted anger, hatred, and other manifestations of distress while undergoing torture but noted that moving forward was essential to sustaining their existence. Three participants reported ongoing struggles with anger and rebellion, frequently channeling these emotions toward those around them. Despite this, they expressed a strong desire to suppress memories of past events, offer forgiveness, and free themselves from the burden of negative emotions. Most participants refused to hold onto resentment, noting that this brought them a sense of liberation and peace. One participant reflected on the power of forgiveness.

*My brother committed suicide because he was unable to forgive.* P6.

Another participant made the following statement:

*If I saw those who tortured me*,* I wouldn’t do anything to them. I would just try to escape from them.* P1.

Rather than seeking revenge or confrontation, this participant’s primary instinct was self-preservation and avoidance, highlighting the deep-seated fear and trauma associated with their tormentors. This suggests that the experience of torture left survivors in a state where the thought of facing their abusers triggered an overwhelming need for safety rather than retaliation. This also underscores the long-lasting emotional scars that made even the idea of encountering their perpetrators unbearable, reinforcing the profound impact of such experiences on their sense of security and agency.

#### Empathy and helping others

Four participants had chosen professions centered on helping others, affirming that this brought a sense of purpose and meaning to their lives. One participant recounted how he spent his leisure time engaging in activities that supported others, particularly those from nations facing significant challenges.

*I am sick now*,* but I really want to recover soon so I can continue my voluntary activities*,* helping others.* P1.

This drive to assist others appeared to serve as a coping mechanism, offering the survivors a sense of fulfillment and purpose, counterbalancing the negative memories and lasting pain from their experiences of torture. One participant recounted his engagement in human rights advocacy with the aim of helping others avoid the sense of injustice that he had experienced.

*I don’t like what is happening in the world*,* so the best way I can help is by staying engaged and taking action.* P6.

This could be interpreted as a way for survivors of torture to reclaim a sense of control and meaning in their lives. By dedicating themselves to caregiving or helping others, they may be seeking to counterbalance the feelings of powerlessness and dehumanization they endured. This renewed sense of purpose can act as a form of healing, allowing them to transform their suffering into something positive and constructive for both themselves and their communities.

#### Physical activity

Four participants described dedicating significant time to physical activity, such as dancing and swimming, to ease the physical effects of torture and divert their attention from their memories.

*While engaging in dancing*,* I feel free! Interacting with others helps me escape from the torture.* P5.

Dancing provided a sense of liberation and emotional relief for the participant. Social interactions and physical expression may serve as a means of escaping the lingering psychological impact of torture, offering a temporary reprieve and fostering a sense of freedom and connection. The heightened receptiveness and reduced seclusion connected to physical activity played a vital role in rebuilding trust and cultivating connections.

One participant spoke at length about how, as a volunteer, he helped many refugees overcome their fears by encouraging them to confront their apprehensions about drowning.

*They are very afraid when they start*,* but I see them gradually become free from their fear. It is a wonderful and transformative journey.* P4.

This can be interpreted as an example of the participant using their volunteer role to support refugees in addressing fear stemming from traumatic experiences, such as perilous journeys across water. By guiding them to confront these fears, the participant helped them regain confidence and emotional resilience. This underscores the transformative power of compassion and support in helping others heal from trauma and regain a sense of safety and control over their lives. It also appears that social integration is essential for the mental and emotional healing of survivors, as it enables them to navigate the aftermath of trauma and rebuild their lives through physical activity and the cultivation of positive relationships.

## Discussion

In this study, participants reflected on the profound effects torture had on their lives, particularly on relationships with others, their bodies, and their strategies for coping. These experiences align with findings from other research, such as [45], who explored the experiences of nine torture survivors and highlighted the complex relationship between torture, pain, re-experiencing trauma, and other facets of individual experience, including profound losses. In Taylor’s study, pain was often described as “the enemy,” emphasizing its intensity, ferocity, and the relentless nature of trauma symptoms that resurface in survivors’ lives [45]. Both Taylor’s findings and ours underscore the deeply embodied nature of trauma from torture, reflecting Merleau-Ponty’s philosophy of the lived body [21]. Torture survivors describe how trauma is not only a psychological experience but also a deeply physical one in which the body itself becomes a site of pain, memory, and re-experiencing. This embodied perspective provides critical insights into the lasting effects of torture and highlights the importance of addressing both the physical and psychological dimensions of healing for survivors.

The application of Merleau-Ponty’s theory in understanding torture experiences and their consequences expands traditional psychological models of trauma by integrating the concepts of perception and embodiment [21, 46]. This approach enables a comprehensive understanding of how torture impacts survivors, not only mentally but also physically and perceptually. By bridging these dimensions, this study enhances existing theoretical frameworks in trauma psychology, such as the neurobiological framework [47] and the cognitive-behavioral framework [48], offering novel insights and establishing a pathway for future research and therapeutic innovations. Other studies that support this perspective in relation to traumatic experiences have also utilized Merleau-Ponty’s theory of the lived body to explore post-traumatic stress disorder (PTSD), emphasizing that trauma is not merely a psychological experience but one that is deeply rooted in the body and often manifests through physical sensations and disruptions in bodily awareness [49, 50]. McDonald’s research, drawing on Merleau-Ponty, highlights the need for a comprehensive understanding of PTSD that integrates phenomenological philosophy with psychology and neuroscience, treating these fields as complementary rather than conflicting [49]. This integrative approach underscores the significance of embodiment in understanding and addressing the complexities of trauma.

In this study, Merleau-Ponty’s phenomenological approach contributes to clarifying the subjective experience of trauma as a consequence of torture, offering a nondualistic view of embodiment, recognizing individuals as inherently adaptive, and highlighting that trauma disrupts the coherence of embodied experiences rather than directly impacting the body or mind. This framework was used in a stydy [51] to explore how survivors of violence or abuse experience disconnection from their bodies and the world around them, emphasizing the body’s central role in perceiving and processing trauma. The study posits that the relationship between violence and embodiment arises from the fundamental role the body plays in shaping our understanding of the world, and violence disrupts and destroys this embodied role, undermining the body’s capacity—often described as the “I can”—to make sense of and engage with the physical, cultural, and social dimensions of our environment [51]. This approach can be directly connected to the experience of torture, in which violence disrupts the body’s fundamental role in making sense of the world. Torture shatters the body’s capacity—the embodied “I can”—to navigate and relate to its environment. By undermining the body’s ability to engage with the physical, cultural, and social significance of its surroundings, torture dehumanizes and disorients survivors, leaving them disconnected from their own sense of agency and meaning. This destruction of the body’s integrative role highlights how torture transcends physical harm, creating profound psychological and existential impacts that redefine the survivor’s relationship with themselves and the world.

By focusing on the lived body, researchers have illuminated how trauma profoundly affects the way individuals inhabit their bodies and relate to their environments, providing a more holistic perspective on the intricate interplay between the physical and psychological dimensions of trauma [29, 52]. This perspective offers valuable insights into recovery processes, highlighting the critical role of reconnecting with the body as an integral part of healing. In doing so, it challenges traditional dualistic views in psychology and other sciences, which often separate the mind and body, emphasizing the need for integrative and embodied approaches to understanding and treating trauma [53, 54]. This approach underscores how the body’s lived experience is central to understanding the impact of torture-related trauma. Torture disrupts the survivor’s relationship with their own body and environment as the body becomes a site of intense suffering and betrayal. This can manifest as a fractured sense of self, alienation from the body, and difficulty re-establishing trust in one’s surroundings [55, 56]. By focusing on the lived body, this approach highlights the necessity of addressing both the physical and psychological dimensions of recovery, offering an integrative framework for healing.

Reconnecting with the body through practices such as somatic therapy, mindfulness, or movement-based therapies can help survivors regain a sense of agency and coherence [57]. This challenges traditional dualistic models that separate physical and mental health and instead advocates for an embodied understanding of trauma [58]. This explanation also sheds light on retraumatization during treatment by highlighting the disruption torture causes to the survivor’s sense of embodiment and agency. Torture undermines the body’s ability to engage meaningfully with its environment, leaving survivors feeling disoriented, dehumanized, and disconnected from their sense of self and the world around them. During treatment, these disrupted connections can resurface when survivors are asked to recount or process their traumatic experiences [59]. Therapeutic settings may inadvertently trigger feelings of vulnerability, powerlessness, or alienation that echo the original trauma, particularly if survivors feel a lack of control, are overwhelmed by sensory or emotional cues, or perceive the therapeutic process as invasive.

Additionally, the survivor’s fractured relationship with their body may make somatic-based therapies or physical sensations during treatment particularly challenging [11]. These experiences can feel like a repetition of the harm rather than a step toward healing, especially if care providers are not attuned to the survivor’s need for safety, agency, and a paced, empathetic approach [15, 60, 61]. This highlights the importance of trauma-informed care, which prioritizes creating a sense of safety, control, and empowerment to avoid retraumatization and foster healing [57, 62].

The four themes of this study (resisting torture–ignoring the body; fear and vulnerability–the unsafe body; broken trust–the broken body; and resilience–rebuilding the body) reveal not only the survivors’ immense trauma but also their strength and courage. The results are consistent with those of other studies of torture experiences involving various torture techniques and a reality of violence and depravity that is typically beyond our capacity to fully understand [63, 64]. The narratives examined in this study are in line with observations in a study [65], discussing how the psychological wounds inflicted on tortured individuals extend beyond the individual to affect the collective social body. The authors argued that torture not only damages the survivor’s sense of self and agency but also disrupts their relationships, social connections, and cultural identity. The trauma inflicted on one person ripples outward, creating fractures in families, communities, and broader societal structures.

In this study, the participants’ narratives revealed a profound impact not only on their individual psychological and physical well-being but also on their ability to maintain and rebuild connections within their social environments. Calvero and Hilde’s observations highlight the interdependence between individual suffering and communal well-being, suggesting that the trauma of torture reverberates throughout the social fabric, affecting collective resilience and cohesion. This perspective helps frame torture as a disruption to the interconnected web of social and cultural relationships.

The torture practices outlined in this study draw attention to violations of global human rights norms condemned by international treaties and conventions [66, 67]. Our study presents testimonies that demonstrate a conspicuous and intentional disregard for these standards. This raises concerns regarding the accountability of perpetrators and the imperative for international oversight. In line with other research, this study highlights the significance of exposing the stories and giving survivors a voice to promote global awareness of torture and prevent its recurrence [68, 69].

In light of Merleau-Ponty’s philosophy, the participants’ narratives are considered embodied experiences that isolate them from the rest of the world [21]. Our bodies connect us to the world [32], and their disruption through torture severs this vital link [70] and impedes survivors’ engagement with their environment and the people who are important to them. This is evident in our participants’ reports that they could not even share their experiences with their own families, highlighting the deep isolation and disconnection inflicted by such trauma. The painful emotions associated with this contributed to the intensification of embodied pain following torture. This aligns with Merleau-Ponty’s [35] emphasis on the profound role of embodiment in shaping the perception of torture experiences [71]. Merleau-Ponty rejects the empiricist interpretation of sensation as a mere response to external stimuli and instead argues that perception is inherently tied to embodied experience, emerging through the lived interplay between the body and the world [72]. Pain’s physical and emotional dimensions are deeply interconnected, making the embodied experience of torture profound and complex, as described by the participants in our study. The pain from torture significantly impacted their bodies and perceptions, creating a heightened and altered awareness of the body driven by the intensity of the pain. Merleau-Ponty’s philosophy explains how perception is influenced by the body, highlighting the need for healthcare providers to understand the physical and psychological consequences of torture to meet the special healthcare needs of survivors [13]. As we interpret Merleau-Ponty, his texts eliminate reductionist tendencies and address the lack of emphasis on preventive measures in a clinical context by utilizing a Cartesian concept of embodiment. By focusing on the unity, purposefulness, and “enworldment” of the lived body, clinical practice can be reoriented to better understand the significance of the body and embodiment in the experience and reactivation of trauma from torture within a healthcare setting [70].

### Resisting torture—ignoring the body

The participants in the study described using mental techniques to dissociate from their physical pain during torture. Some participants spoke about mentally “leaving” their bodies or focusing on specific memories, imagined safe spaces, or abstract thoughts to detach themselves. This enabled them to endure extreme suffering as they viewed their bodies as separate from their sense of self. This form of detachment acted as a crucial survival mechanism, allowing them to preserve their identity and resilience in the face of severe physical and psychological trauma. Drawing on Merleau-Ponty’s phenomenology, which emphasizes the significance of the “lived body” in shaping our experiences and interactions with the world, we introduced the term “ignoring the body” to describe the participants’ strategies for enduring torture.

Consistent with other studies, the participants reported heightened physiological responses, chronic pain, and somatic symptoms [10]. These physical manifestations reflected their embodied experiences, in which the body became a site of suffering and mistrust [21]. Other studies have also described hypervigilance, muscle tension, and stress responses in healthcare settings that evoke memories of torture and cause retraumatization [18]. According to Merleau-Ponty’s theory of the lived body, these reactions are deeply rooted in survivors’ embodied experiences. This perspective offers critical insight into retraumatization, framing it as more than a solely psychological phenomenon. It underscores how trauma is rooted in the body, with survivors’ physical responses reflecting their lived experiences and highlighting the importance of integrative approaches to healing. This has significant implications for psychologists and healthcare providers, emphasizing the need for trauma-informed, body-centered practices. Torture disrupts the body’s natural harmony, forcing survivors into a state of traumatic hyper-awareness as they focus on enduring inflicted pain, which interrupts the body’s natural functioning. This aligns with Merleau-Ponty’s observation that much of bodily experience is prereflective, occurring without conscious thought [73].

Merleau-Ponty’s view of the body as both physical and perceptual [74] underscores how torture distorts perception, with victims’ efforts to ignore pain reflecting an attempt to counteract this forced hyper-awareness and its accompanying trauma. Survivors of torture may also instinctively suppress or ignore pain during medical treatment as a coping mechanism learned through their traumatic experiences. This automatic response poses challenges in the assessment and treatment of pain. Survivors’ tendency to endure discomfort necessitates regular follow-up to monitor recovery and reveal any unreported pain, ensuring that it does not escalate into more severe issues. Several studies have demonstrated that torture survivors often experience alterations in pain perception and modulation [75, 76], yet in practice, there are few or no adjustments made to improve the quality of pain treatment and tailor it to their unique needs [11].

When addressing pain related to torture, it is important to consider the role of touch, as it plays a fundamental role in caregiving and healing [77]. The participants in our study described their experiences of touch during torture as synonymous with violence and pain, reflecting a deeply negative embodied experience [78]. Healthcare providers must be sensitive to the implications of touch when treating torture survivors. While touch is often a routine part of care [79], for survivors, it can trigger trauma-related associations and increase the risk of retraumatization. Recognizing and addressing the lived body and understanding the importance of touch are essential for improving patient outcomes and enhancing the professional abilities of healthcare providers [80].

Touch plays a crucial role in building connections, allowing us to relate to others as embodied beings. In caregiving, it conveys empathy and compassion, reinforcing human relationships. Merleau-Ponty suggests that touch shapes our perception of the world and ourselves [81, 82]. For trauma survivors, touch can also symbolize harm. In caring for torture survivors, touch must be used with sensitivity to avoid triggering memories of pain and violation [82].

#### Abandonment: the insignificant body

The participants described feelings of abandonment stemming from denial and a lack of recognition of their torture experiences. This denial disrupted intersubjective recognition, stripping survivors of their relational identity, reducing them to objects, and severing their connection with others and their environment. This dehumanizing process fractured their embodied sense of being-in-the-world, leading to isolation, abandonment, and existential despair. Merleau-Ponty’s philosophy of intercorporeality emphasizes the interconnectedness of human bodies and shared experiences, highlighting that our sense of self is rooted in interactions with others and the environment [83]. The participants’ feelings of abandonment left them feeling insignificant to others, thus eroding their trust in people. This fundamentally altered their embodied experience, leading to chronic symptoms, disconnection from bodily sensations, and difficulty inhabiting their bodies. This highlights the importance of restoring intersubjective connections and re-establishing the survivor’s embodied presence within a relational world, including healthcare settings that acknowledge and address the experiences of torture and its consequences.

By addressing the embodied nature of trauma, practices like group therapy and body-centered approaches, aligned with Merleau-Ponty’s intercorporeality, offer pathways for survivors to reconnect with others, rebuild relationships, and reclaim their embodied sense of self [84]. Treating torture is not solely about addressing the survivor’s individual needs but also involves supporting their families and communities. This requires a multifaceted approach, including family therapy to restore relational bonds, community-based interventions to foster collective healing and reintegration, culturally sensitive practices to align interventions with the survivor’s context, and psychoeducation for families and communities to improve the understanding of trauma and provide empathetic support [85]. An example of such an approach was presented in a study [86], which explored the development and feasibility of a multi-couple group intervention for torture-surviving couples in the Democratic Republic of Congo, a community affected by widespread torture during the 1998–2004 wars. Their short-term resource-efficient model aimed to address relational difficulties caused by trauma. Over 10 weekly sessions, 13 couples reported significant improvements in their relationships [86].

#### Hopelessness: longing for release from the confines of the flesh

This study’s participants described how torture caused existential disintegration—the collapse of the self’s unity and coherence—intensifying their longing for an end to their suffering, and even to life itself. They expressed a profound sense of hopelessness and a fragmented sense of existence. This yearning for release underscores the devastating impact of torture on both the embodied self and the survivor’s connection to life and the world. Torture profoundly fractured their sense of self, with intense and uncontrolled pain fragmenting their identity and inducing feelings of disownership and existential disintegration, as described by Merleau-Ponty [87]. Disownership arises when survivors no longer recognize their bodies as their own and experience deep alienation from their physical form. Under such extreme conditions, the body becomes a site of unbearable suffering, leading survivors to long for release from the confines of the flesh, as discussed in Merleau-Ponty’s phenomenology [88].

This reflects a desperate desire to escape the physical pain and trauma that tie their identity to a violated body. Merleau-Ponty emphasizes the body as central to our experience of being-in-the-world, yet torture disrupts this harmony and transforms the body into a prison of suffering.

To address the physical and existential impacts of torture, including this longing for release from the flesh [87], treatment should prioritize reconnecting the survivor’s body and sense of self. Noninvasive techniques that promote bodily awareness without triggering discomfort, empowering survivors to maintain control over their treatment, and creating a calm, predictable environment free from triggers such as sudden movements or invasive procedures are key strategies for enhancing quality of care, as suggested in earlier studies [16, 61, 89].

#### Protecting others: offering their own body

The participants in this study described offering their bodies to shield others, exemplifying an embodied existence in which the body becomes a site of ethical action. This reflects a recognition of shared humanity and a profound ethical commitment, aligning with Merleau-Ponty’s framework of relational being [88], which describes the body as a lived experience that shapes our interactions with the world and others [32, 90]. Ethical actions, as noted in a study [91] exploring corporeal ethics, are deeply embodied, with the pain and suffering of others felt viscerally, prompting selfless acts grounded in our bodily engagement with the world. This perspective may also explain why torture survivors tend to protect healthcare providers who fail to meet their treatment needs [11]. They may attribute shortcomings to systemic issues rather than individual failings, as illustrated in a study exploring torture survivors’ experiences of receiving surgical care. The study described how survivors were offered local anesthesia instead of general anesthesia for surgical procedures, despite the known challenges torture survivors face with pain [92]. Healthcare providers must deliver compassionate care while advocating for systemic changes that ensure survivors receive appropriate, trauma-sensitive treatment. Understanding this can foster greater accountability and support the development of trauma-informed healthcare systems.

#### Betrayal: the denied body

This study’s participants described profound betrayal—often by friends and family—that intensified their disconnection from their own bodies as self-protection from feelings of disappointment. This betrayal compounds the physical and emotional harm inflicted during torture, as survivors experience their relationships as sources of deep mistrust. The double betrayal—by both their social network and their own embodied existence—further alienates survivors from the world and themselves.

As an embodied experience, betrayal exacerbates the tendency to dissociate from the body as a coping mechanism. Survivors may deny their physical sensations and emotional pain to protect themselves from the overwhelming reality of being harmed by those they once trusted. The lived body becomes an object of suffering, disconnected from their sense of self and relational identity [21].

This estrangement presents unique challenges in treatment. Survivors may replicate denial reactions as a protective mechanism, such as dismissing pain or rejecting a diagnosis, to shield themselves from vulnerabilities rooted in both their physical and relational trauma. Addressing this requires a dual focus: helping survivors reconnect with their bodies while also rebuilding trust in relationships, emphasizing safety and validating their experiences of betrayal. Only by addressing both the embodied and relational aspects of their trauma can healing and reintegration begin.

Trauma-informed therapeutic approaches, such as somatic therapies, can help survivors gradually rebuild trust in their bodies and integrate their embodied experiences into a renewed sense of self. Recognizing the body as both a site of trauma and a potential source of healing is crucial in supporting survivors on their recovery journey. By understanding the denial of bodily experiences as a protective strategy, healthcare providers and therapists can approach survivors with greater empathy, enabling them to create safe and compassionate environments where survivors feel empowered to reconnect with their embodied selves at their own pace [56]. Consistency and transparency, predictable interactions, active listening, respecting bodily boundaries, and a nonconfrontational approach can significantly enhance the quality of treatment for survivors of trauma. These strategies foster a sense of safety, build trust, and create an environment where survivors feel understood and supported in their healing journey [16].

#### Silence: the invisible body

The participants in this study described struggling to maintain silence under torture, balancing the need to withhold information for survival with a deep yearning for recognition and support. This tension reflects a fragmentation of the self, as the natural human drive for communication conflicts with the imperative of self-preservation. Viewed through Merleau-Ponty’s emphasis on the embodied and relational nature of human experience, this dynamic highlights how torture disrupts the connection between self, others, and the world. The long-term psychological impact of suppressing one’s voice exacerbates isolation and alienation, underscoring the need for therapeutic spaces where survivors can safely reconcile silence and expression to rebuild their sense of self. The struggle described by participants—to balance silence and the yearning for recognition—connects deeply to the reality that healthcare providers often avoid asking about torture [11, 16]. This silence in healthcare settings perpetuates the survivor’s isolation as their experiences remain unacknowledged. The natural human drive for communication and validation, which torture disrupts, remains unmet when providers fail to create spaces for survivors to share their stories. Acknowledging torture is important for helping survivors navigate the psychological fragmentation caused by their experiences. If kept in silence, survivors may feel that their suffering is unimportant or shameful, reinforcing their alienation and disconnection.

Silence within society and their families was described by participants as exacerbating their feelings of isolation, emotional distress, shame, and guilt, and connects to the metaphor of the “ignored body.” For torture survivors, silence involves not only mental effort but also significant physical struggle. During and after torture, instinctive reactions to pain and the need to communicate are suppressed, leading to a sense of being ignored and unacknowledged by society and disrupting interactions with the world [71]. When survivors withhold their experiences, they suppress deeply ingrained narratives, leading to isolation and melancholy. While this suppression is often motivated by a desire to shield loved ones from the horrors of their past, it can inadvertently cause significant emotional distress. Survivors may experience guilt and sadness for not revealing their true experiences, as this silence creates a barrier to authentic connection and prevents them from fully processing their trauma [93]. The emotional burden of withholding such narratives may manifest as psychosomatic symptoms or exacerbate existing conditions. Healthcare providers might struggle to address these issues without understanding their root cause. The emotional distance created by survivors’ silence can hinder the development of a therapeutic alliance, making it challenging for healthcare providers to establish trust and provide holistic care [55].

### Fear and vulnerability—the unsafe body

From Merleau-Ponty’s perspective [94], fear arises from the body’s response to suffering, aiming to prompt protective actions. The participants described intense fear during torture, driven by uncertainty, a lack of control, and the inability to protect themselves, leading to an overwhelming sense of vulnerability. During medical procedures like surgery, survivors may have similar feelings of vulnerability and an inability to act protectively [11, 61]. Although surgery aims to heal, it requires the surrender of agency, turning the body into a passive object. This can split the self: one part strives to maintain coherence, while the other dissociates as a protective mechanism, leading to estrangement from the body, now perceived as “for others” [95]. Protective mechanisms may also be essential in clinical settings, particularly because feelings of fear during medical treatment are often closely linked to survivors’ past experiences of torture. In these situations, survivors may find themselves unable to take protective actions to shield themselves, reinforcing a deeply ingrained sense of helplessness. This embodied memory of helplessness can resurface during medical procedures, where survivors may once again lose control over their body [12, 89].

Other conditions of medical treatment—such as being restrained, anesthetized, or subjected to invasive procedures—can replicate the physical and psychological dynamics of torture, potentially triggering a re-experiencing of the trauma [11, 61]. Implementing protective mechanisms, such as clear communication, patient involvement in decision-making, and creating a sense of safety, has been recognized as crucial [16, 61]. These strategies can help reduce fear and restore a degree of control and agency, preventing further harm and enabling a more supportive and healing clinical experience for survivors.

Healthcare providers must recognize that survivors of torture may perceive medical procedures as situations that replicate feelings of vulnerability and powerlessness. Addressing this requires careful communication, trust, and an environment where the survivor feels safe, respected, and involved in their care. Such approaches help mitigate the risk of retraumatization and allow survivors to regain a sense of agency over their body and experience.

Recovery, according to Merleau-Ponty, involves restoring the body’s agency and reconnection to the world. For torture survivors, this means relearning to trust their body and environment. Healthcare providers must foster safety, agency, and respect through clear communication, consent-driven care, and collaborative treatment. Offering survivors control—whether in medical care or therapy—helps counteract the loss of agency experienced during trauma [11, 89]. Studies have shown that regaining control fosters trust in the body, environment, and relationships [96], promoting empowerment, resilience, and long-term recovery [97].

#### Fear and vulnerability related to their own suffering: the alerted body

The participants in this study described profound feelings of vulnerability during and after their torture experiences, which can be understood through the concept of the “alerted body.” Torture disrupts the fundamental sense of bodily safety and integrity, leaving survivors in a constant state of alertness and insecurity. When bodily safety is compromised, it profoundly impacts self-perception and relationships [98].

For torture survivors, the body becomes a site of trauma and fear, perpetually in a state of heightened alertness [93, 99]. Routine healthcare interactions—such as physical exams, injections, or invasive procedures—can easily trigger these feelings if healthcare providers do not create a safe and supportive environment. The issue is exacerbated when providers fail to recognize or address embodied trauma experiences, intensifying their sense of insecurity and risking retraumatization [80]. This heightened state of alertness was also noted in a study on dentistry involving torture survivors, in which participants reported strong reactions to stimuli. This underscores the importance of creating a safe therapeutic space and prioritizing the patient’s sense of control [61]. Healthcare providers must take steps to address the heightened vulnerability and “alerted body” state in torture survivors, ensuring that care is trauma informed and creating a safe and predictable environment [11, 89].

#### Fear and vulnerability related to others’ suffering: the unstable body

The participants in our study recounted episodes during captivity in which they feared for the lives of others and suffered deeply from witnessing the torture of others, describing this as even more difficult to endure than their own torture. The concept of “the unstable body” aligns closely with the fear and vulnerability tied to witnessing others’ suffering. From Merleau-Ponty’s perspective, the body is a lived, relational experience deeply connected to others through intercorporeality [100]. Witnessing others being tortured disrupts this natural connection, leaving survivors’ bodies in a heightened state of alertness and instability.

When survivors viscerally respond to the suffering of others, their bodies echo the pain as if it were their own [101]. This creates an unstable bodily experience, where the boundaries between self and others blur, embedding the trauma of witnessing into their own physical and emotional being. This empathetic resonance becomes a source of ongoing vulnerability as the survivor’s body remains in a constant state of tension, reliving both personal and observed trauma. The unstable body reflects this perpetual state of alertness, in which the survivor is unable to fully ground themselves in safety or a coherent sense of bodily autonomy.

This instability amplifies the disrupted relationship survivors have with the world, where their bodies, instead of being sources of agency and connection, become mediums of relived fear and suffering. The survivor’s heightened sensitivity to stimuli and difficulty distinguishing between their own pain and others’ suffering perpetuates this unstable state. This dynamic becomes particularly visible when survivors accompany family members to medical treatment, as they may experience retraumatization if they perceive their loved ones receiving inappropriate care. Witnessing what they interpret as neglect, lack of empathy, or procedural errors can evoke memories of past betrayals and harm, reactivating the embodied trauma of witnessing others’ suffering during torture.

From the perspective of “the unstable body,” this reaction highlights how the survivor’s empathetic connection to others’ pain remains deeply embodied. Their body responds viscerally to the distress of family members, mirroring the emotional and physical toll of their own past experiences. The survivor’s heightened alertness and vulnerability make them particularly sensitive to any signs of perceived harm, even in routine medical contexts.

#### Guilt and shame: the ashamed and guilty body

The participants in this study disclosed profound feelings of shame and remorse derived from their experiences of torture and its aftermath. Numerous accounts of profound humiliation were linked to their personal suffering, frequently accompanied by a feeling of helplessness during their ordeal. Furthermore, they conveyed profound remorse—not only for the perceived influence their experiences had on their families and friends but also for the anguish they observed in others. Witnessing the torture of others, particularly comrades or fellow detainees, frequently exacerbated these emotions as participants grappled with feelings of powerlessness and a sense of complicity for being unable to intervene. The belief that their loved ones bore emotional, psychological, or material burdens as a consequence of their circumstances further exacerbated these emotions. This underscores the multifaceted and relational aspects of the psychological aftermath of torture as survivors contend with the burden of both personal and vicarious trauma. Survivors often internalize these traumas, with shame rooted in the violations they endured and guilt stemming from their perceived inability to resist or to protect others [21, 102]. This aligns with Womersley’s (2019) reflections on shame in torture survivors, highlighting its detrimental impact on health-seeking behavior. Shame often manifests subtly, going unnoticed by practitioners [103]. This poses a significant challenge for clinicians and researchers working with refugee populations, where power dynamics and intersecting identities influence not only survivors’ experiences of shame but also those of the clinicians and researchers themselves.

Medical procedures, especially those involving undressing, physical touch, or invasiveness, can trigger this embodied shame and guilt, reactivating trauma and leading to retraumatization [12]. These triggers can deepen survivors’ distress, impeding their ability to engage with care. Shame also disrupts the survivor’s relational world as their connection to others becomes fractured, and guilt amplifies feelings of failure, particularly when they believe they failed to protect others. This embodied shame often manifests as tension, withdrawal, or hyper-awareness of others’ perceptions, altering the survivor’s sense of being-in-the-world.

Torture often leaves visible scars or disfigurement, which can evoke feelings of shame in survivors. Visible disfigurements, while deeply impactful, are not inherently sufficient to cause shame [104], but the meaning and significance of these scars are shaped within the survivor’s consciousness and closely tied to their self-perceptions. However, survivors have the freedom to reinterpret the meaning of these physical marks and determine the extent to which they allow others’ perceptions to affect their sense of self.

As described by Merleau-Ponty, in some contexts, disfigurements—such as battle wounds or injuries sustained by firefighters—can be reframed as symbols of bravery or badges of honor, reducing the potential for shame or negative societal reactions [104]. For survivors of torture, scars often carry a more complex and nuanced narrative. These marks may evoke societal stigma or personal self-consciousness; however, they can also inspire empathy, admiration, and solidarity.

These external responses are particularly significant in healthcare settings, where compassionate and empathetic care can profoundly influence a survivor’s healing process. Combined with the survivor’s own resilience, these positive reactions can help protect against internalized shame and foster a sense of empowerment. By acknowledging the lived experience of disfigurement and addressing both its physical and emotional dimensions, healthcare providers can support survivors on their journeys toward recovery and self-redefinition. This may also foster trust and minimize retraumatization [80].

### Broken trust—the broken body

The bodies of survivors bear the imprint of their trauma, which affects their ability to trust others [55]. The participants in this study highlighted their struggles with trusting others, stemming from the betrayals they experienced during torture. These betrayals disrupt their sense of safety, making it difficult to form or maintain meaningful relationships. Interactions with healthcare providers can trigger fear and apprehension [105, 106]. Merleau-Ponty’s concept of the “lived body” explains how bodily memories of trauma influence survivors’ perceptions, making it difficult to feel safe even in supportive environments [55, 107]. The participants’ feelings of mistrust are not merely cognitive but an embodied reality, where the body’s reactions and the mind’s perceptions are intertwined. During healthcare interactions, this can lead to retraumatization, with medical procedures evoking sensations and memories of the original trauma, highlighting the complex interplay between psychological and physiological responses in treatment.

The lack of trust often stems from the existential crisis caused by torture. This existential disintegration results in a pervasive sense of mistrust, isolation, and doubt about the integrity of human relationships. The theme of “broken trust,” reframed as the “broken body” through Merleau-Ponty’s theory [21], underscores the profound impact of torture on survivors’ trust in themselves and others. The “broken body” symbolizes survivors’ betrayal, reflecting the loss of trust in their own bodies and human connections [108].

For healthcare providers, understanding “broken trust” as a manifestation of the “broken body” is crucial to avoiding retraumatization. By acknowledging the embodied nature of torture and adopting empathic, respectful, and compassionate treatment, providers can support healing and rebuild trust through kind and thoughtful care of the “broken body” [55, 80]. Prior research has highlighted survivors’ mistrust of healthcare providers [12]. When doctors become complicit in torture, they deeply undermine trust in healthcare, a betrayal that can persist into survivors’ resettlement contexts [56]. Since relationships shape our sense of self and reality [21], this betrayal fosters a pervasive mistrust that defines survivors’ interactions with others, including healthcare professionals.

Creating a nonjudgmental therapeutic space is crucial for rebuilding trust. Research suggests that being nonjudgmentally aware and compassionate toward survivors’ suffering is critical for those repeatedly harmed by others [109]. Survivors need to feel that their emotions—including anger, fear, or mistrust—are respected and validated to facilitate healing.

### Resilience—rebuilding the body

The theme “resilience–rebuilding the body” underscores the critical role of recovery for torture survivors. The participants described actively working to restore their physical, psychosociological, and emotional well-being through physical healing, thereby rebuilding social connections and finding new purpose. Their resilience aligns with studies showing that social relations and support attenuates the effect of torture exposure on PTSD [110]. Clinical approaches must be tailored to individual coping styles when supporting survivors. By understanding and supporting these pathways, practitioners and policymakers can design more effective interventions that harness the innate resilience of torture survivors by fostering environments that encourage altruism, physical engagement, and community building. These strategies not only aid individual recovery but also contribute to collective healing and empowerment.

The concept of “rebuilding the body,” rooted in Merleau-Ponty’s focus on embodiment, offers a profound lens for understanding resilience. From this perspective, healthcare providers can better grasp the impact of torture and identify factors that promote resilience during treatment, helping prevent retraumatization. This approach highlights the importance of trauma-informed care that acknowledges and respects survivors’ embodied experiences. By fostering a compassionate and supportive environment, providers can facilitate holistic healing and empower survivors in their recovery journey.

Forgiveness emerged as a significant strategy for moving forward and rebuilding the body. For some participants, the act of forgiveness represents resilience, enabling them to move beyond past trauma and embrace a hopeful future [93]. According to Merleau-Ponty, forgiveness can be understood as a deeply embodied and relational act. It is not merely a cognitive or abstract decision but an experience rooted in the lived body and our interconnection with others. Forgiveness involves reconciling the disruptions in our embodied relationships caused by harm, betrayal, or trauma. It allows individuals to restore a sense of relationality and reengage with the world and others in a way that heals the fractures in their sense of self and their trust in human connections. For torture survivors, forgiveness, whether directed toward others or themselves, may play a critical role in rebuilding their embodied being-in-the-world and fostering a sense of agency and resilience [111].

The participants also described the importance of helping others as a way to move forward. Such acts allow survivors to channel their suffering into constructive and meaningful activities, reinforcing their sense of humanity and agency [93]. Our own bodily responses to others’ distress, such as flinching or discomfort, highlight the interconnectedness between our physical experiences and empathy. Helping others can elicit positive emotional reactions, counteracting the negative effects of past torture and imprisonment. This reflects Merleau-Ponty’s idea that empathy is rooted in the connectivity of our physiological experiences with those of others [35]. Helping others and engaging in physical activity often work synergistically to promote resilience, as described by the participants. Both practices encourage active participation in life, shifting the focus from past trauma to present agency and future possibilities.

For healthcare providers, understanding the resilience strategies that torture survivors use in daily life can guide adjustments in the clinical setting, enabling survivors to prevent retraumatization and reclaim agency over their experiences. Relating this to Merleau-Ponty’s philosophy, the body is central to perception and experience, and resilience strategies—such as prayer—are deeply embodied practices that provide meaning and stability. Prayer, involving posture, voice, or ritual movements, serves as an anchor for survivors and helps them navigate their lived reality. By recognizing and respecting such practices, providers can create a clinical environment aligned with the survivor’s bodily and perceptual needs, fostering holistic healing. Additionally, integrating opportunities for physical rehabilitation, social engagement, or altruistic activities within healthcare settings can reinforce coping mechanisms and promote empowerment.

### Retraumatization

Merleau-Ponty’s phenomenology underscores how the healthcare environment and its sensory cues significantly shape recovery, as care is experienced through the survivor’s embodied perceptions [80].

Survivors’ bodies retain the imprints of pain and suffering, profoundly shaping their interactions and reactions long after the events [10]. Encounters with environments, sounds, or physical sensations reminiscent of their trauma can cause survivors’ bodies to react as though they are reliving the traumatic event [15]. This embodied memory manifests through physical symptoms, heightened emotional responses, and visceral reactions, which can make healthcare settings feel unsafe and overwhelming [9].

The societal and familial silence surrounding torture often extends into healthcare settings, where providers may avoid discussing torture or fail to recognize its significance. This avoidance mirrors the conditions of torture, where the survivor’s suffering is rendered invisible or invalidated, reinforcing feelings of neglect and retraumatization [12]. In re-traumatization, the body’s automatic responses, rooted in trauma, can overshadow rational understanding, collapsing the distinction between the past and present. Survivors may experience intense distress in response to routine procedures or interactions, making it difficult to feel safe. Merleau-Ponty’s concept of the lived body highlights that habitual bodily actions shape our sense of self and connection to the world, but torture disrupts these habits [112]. In healthcare contexts, these disruptions can resurface, and survivors may struggle with feelings of disorientation and mistrust. Understanding trauma as an embodied experience ensures that interactions avoid replicating the dynamics of torture and instead foster safety, trust, and recovery.

### Strengths and limitations

The qualitative design allowed for an in-depth exploration of the nuanced and complex experiences of torture survivors. By focusing on the participants’ personal narratives, the study captured rich, detailed insights that might be missed in quantitative approaches. The use of in-depth interviews facilitated a thorough examination of the participants’ lived experiences, enabling the researchers to explore their emotional, psychological, and embodied responses to trauma. This method provided a platform for survivors to voice their experiences in their own words, offering a powerful and authentic representation of their realities.

The study included participants from diverse linguistic and national backgrounds. This enriched the analysis and allowed for broader insights into the shared and unique aspects of survivors’ experiences. The thematic analysis provided a structured approach to identifying and interpreting key patterns in the data, while maintaining the flexibility to explore the individual stories of each participant. This balance between thematic consistency and personal depth strengthens the study’s credibility and relevance.

The study involved six participants, which, while sufficient for in-depth qualitative exploration, limits the generalizability of the findings. The small sample size may not capture the full diversity of experiences and perspectives of torture survivors across different contexts and backgrounds.

The use of thematic analysis, while well-suited for exploring patterns and themes within qualitative data, is inherently interpretive. The findings may have been influenced by the researchers’ perspectives and choices during the data coding and theme development. While every effort was made to ensure rigor and reliability, alternative interpretations of the data were possible.

The study used Merleau-Ponty’s existential phenomenology as the primary theoretical framework to guide the discussion. While this approach provided deep insights into the embodied and relational aspects of trauma, it is one of many potential frameworks that could be applied. The reliance on a single framework may limit the scope of the analysis, particularly when addressing aspects of trauma that might be better understood through alternative psychological or sociological theories.

Participants were recruited from healthcare facilities, which may have influenced the nature of their accounts. For example, the focus on healthcare settings might have emphasized medicalized aspects of their experiences, potentially underrepresenting other dimensions of their lives, such as socio-political or economic impacts.

The interviews were conducted in Norwegian, with one requiring the use of an interpreter. While care was taken to maintain the integrity of the data during translation and analysis, there is always a risk of losing nuance or meaning, particularly when dealing with complex and emotionally charged topics [113]. The use of IPA in this study provided a robust methodological approach for examining the subjective experiences of torture survivors and allowed a detailed and nuanced understanding of how individuals make sense of their traumatic experiences.

### Implications for practice

This study, grounded in Merleau-Ponty’s existential-phenomenological framework, offers several important implications for clinical and healthcare practices aimed at supporting survivors of torture. The integration of Merleau-Ponty’s framework encourages healthcare providers, including psychologists, to move beyond a purely psychological understanding of trauma. Recognizing the embodied nature of trauma means acknowledging how torture impacts both the physical and mental dimensions of survivors’ lives.

Practitioners should address the interconnectedness of the mind and body, incorporating somatic therapies and interventions that help survivors reconnect with their bodies in safe and empowering ways. Given the profound impact of torture on a survivor’s ability to trust and engage with the world, healthcare providers must create environments that are safe, empathetic, and supportive.

Trauma from torture often disrupts the survivor’s basic sense of safety and trust, particularly in institutional contexts. Healthcare providers can foster recovery by cultivating consistent and compassionate relationships that help survivors rebuild a sense of trust in others. Ensuring that care processes respect survivors’ autonomy and preferences empowers them to take an active role in their recovery.

The study underscores the importance of recognizing the risk of retraumatization in healthcare settings, particularly where medical instruments or procedures might echo past torture experiences. Training healthcare professionals in trauma-informed practices is crucial for mitigating these risks.

Merleau-Ponty’s concept of the lived body emphasizes how survivors experience the world through their bodies, even when the trauma of torture has altered this relationship. This perspective encourages practitioners to consider how survivors’ lives have been transformed by their experiences. Interventions must be developed that help survivors re-establish a positive connection with their physical selves and the external world.

## Conclusion

By integrating Merleau-Ponty’s existential-phenomenological framework, this study introduces a philosophical perspective that enriches the understanding of trauma from torture. This integration encourages psychologists and other healthcare professionals to adopt a more holistic view of trauma, considering both the mental and bodily dimensions of survivors’ experiences.

Merleau-Ponty’s phenomenology helps us understand how the physical and psychological impact of torture can persist, affecting a survivor’s ability to engage with the world and with others, even in the context of healthcare. Healthcare providers must recognize that addressing psychological trauma alone is insufficient; they must also consider the embodied nature of trauma. Creating a safe, empathetic, and supportive environment can help mitigate these integrated bodily responses, allowing survivors to gradually rebuild a sense of trust and safety.

The experiences of individuals who have survived torture serve to increase awareness regarding the widespread occurrence and far-reaching consequences of torture. Through the act of recounting their experiences, survivors contribute to the comprehension of the tangible and mental consequences of torture for others. Merleau-Ponty’s perspective on the lived body offers a different comprehension of the connection between the body and the external world, providing an additional dimension to the care of the tortured body within healthcare and the prevention of retraumatization. This understanding has the potential to influence public opinion, the formulation of policies, and global endeavors aimed at preventing torture, as well as providing assistance and support and enhancing healthcare services for survivors.

## Electronic supplementary material

Below is the link to the electronic supplementary material.

Supplementary Material 1

Supplementary Material 2

Supplementary Material 3

## Data Availability

The datasets obtained and/or analyzed during the current study are stored and can be available from the corresponding author (ACS) on reasonable request.
