# Supplementary material for: “After torture, everything changed”: the unpacking of trauma from torture with interpretative phenomenological analysis and Merleau-Ponty’s theory of the lived body
Source: BMC Psychol. 2025 Mar 9;13:219. doi: 10.1186/s40359-025-02507-4 (PMC11890556; doi:10.1186/s40359-025-02507-4)
Supplement: Supplementary file 1 — Supplementary Material 1 [file 40359_2025_2507_MOESM1_ESM.docx]

**Supplemental file 2: Interview guide**

| Semi-structured interview | |
| --- | --- |
| 1. Torture history and methods | Could you please tell me about your background? What were your previous experiences before coming to Norway? |
|  | Please tell me what you think are the most hurtful or scary things that have happened to you. Please tell me when and where these things happened. |
|  | What is the worst thing that has happened to you in your current living situation (i.e., refugee camp, country of resettlement, returned from exile, etc.) if it is not one of the things listed above? Please tell me where and when these things took place. |
|  | Are there any other details you'd like to share about this topic? |
| 1. Hypersensitivity (distrust, vulnerability, powerlessness, loss of control, low self-esteem, and difficulty self-advocating) | Has your trust in other people changed as a result of the torture? Can you tell which way it is? |
|  | Do you think your experiences in your home country have made you more sensitive? In what sense? |
|  | Do you think your self-esteem has changed as a result of the torture? In what way? |
|  | Do you think torture has made it more difficult to make decisions? |
|  | Are there other things about this subject you want to tell? |
